# Very mild disease phenotype of congenic *Cftr*^*TgH(neoim)Hgu *^cystic fibrosis mice

**DOI:** 10.1186/1471-2156-9-28

**Published:** 2008-04-09

**Authors:** Balázs Tóth, Martina Wilke, Frauke Stanke, Martina Dorsch, Silke Jansen, Dirk Wedekind, Nikoletta Charizopoulou, Alice Bot, Marion Burmester, Sabine Leonhard-Marek, Hugo R de Jonge, Hans-Jürgen Hedrich, Gerhard Breves, Burkhard Tümmler

**Affiliations:** 1 Physiologisches Institut, Stiftung Tierärztliche Hochschule Hannover, Bischofsholer Damm 15/102, D-30173 Hannover, Germany; 2 Klinische Forschergruppe, OE 6710, Medizinische Hochschule Hannover, Carl-Neuberg-Strasse 1, D-30625 Hannover, Germany; 3 Department of Biochemistry, Erasmus University Medical Center, PO Box 1738, 3000 DR Rotterdam, The Netherlands; 4 Zentrales Tierlaboratorium, OE 8600, Medizinische Hochschule Hannover, Carl-Neuberg-Strasse 1, D-30625 Hannover, Germany

## Abstract

**Background:**

A major boost to cystic fibrosis disease research was given by the generation of various mouse models using gene targeting in embryonal stem cells. Moreover, the introduction of the same mutation on different inbred strains generating congenic strains facilitated the search for modifier genes. From the original *Cftr*^*TgH(neoim)Hgu *^mouse model with a divergent genetic background (129/Sv, C57BL/6, HsdOla:MF1) two inbred mutant mouse strains CF/1-*Cftr*^*TgH(neoim)Hgu *^and CF/3-*Cftr*^*TgH(neoim)Hgu *^had been generated using strict brother × sister mating. CF/1-*Cftr*^*TgH(neoim)Hgu *^and CF/3-*Cftr*^*TgH(neoim)Hgu *^mice were fertile and showed normal growth and lifespan. In this work the *Cftr*^*TgH(neoim)Hgu *^insertional mutation was backcrossed from CF/3-*Cftr*^*TgH(neoim)Hgu *^onto the inbred backgrounds C57BL/6J and DBA/2J generating congenic animals in order to clarify the differential impact of the *Cftr *mutation and the genetic background on the disease phenotype of the cystic fibrosis mutant mice. Clinical and electrophysiological features of the two congenic strains were compared with those of CF/1-*Cftr*^*TgH(neoim)Hgu *^and CF/3-*Cftr*^*TgH(neoim)Hgu *^and wild type controls.

**Results:**

Under the standardized housing conditions of the animal facility, the four mouse strains CF/1-*Cftr*^*TgH(neoim)Hgu*^, CF/3-*Cftr*^*TgH(neoim)Hgu*^, D2.129P2(CF/3)-*Cftr*^*TgH(neoim)Hgu *^and B6.129P2(CF/3)-*Cftr*^*TgH(neoim)Hgu *^exhibited normal life expectancy. Growth of congenic cystic fibrosis mice was comparable with that of wild type controls. All mice but D2.129P2(CF/3)-*Cftr*^*TgH(neoim)Hgu *^females were fertile. Short circuit current measurements revealed characteristic response profiles of the HsdOla:MF1, DBA/2J and C57BL/6J backgrounds in nose, ileum and colon. All cystic fibrosis mouse lines showed the disease-typical hyperresponsiveness to amiloride in the respiratory epithelium. The mean chloride secretory responses to carbachol or forskolin were 15–100% of those of the cognate wild type control animals.

**Conclusion:**

The amelioration of the clinical features and of the basic defect that had emerged during the generation of CF/3-*Cftr*^*TgH(neoim)Hgu *^mice was retained in the congenic mice indicating that the *Cftr *linkage group or other loci shared between the inbred strains contain(s) the major modifier(s) of attenuation of cystic fibrosis symptoms.

## Background

Cystic fibrosis (CF) is a severe monogenic disorder of ion transport in exocrine glands [1]. Mutations in the *CF transmembrane conductance regulator *(*CFTR*) gene lead to impaired epithelial chloride secretion. Dehydration and plugging of mucous secretions in the ducts of exocrine glands predispose to multiorgan clinical manifestations, particularly in the gastrointestinal, hepatobiliary, reproductive and respiratory tracts. In the intestine the chloride and fluid secretion are impaired while sodium and sodium-linked nutrient absorption are enhanced. The resulting dehydration of luminal contents contributes to many of the gastrointestinal manifestations of CF, including the meconium ileus of the neonate [1].

Several mouse models of CF were generated by gene targeting approaches whereby either the murine *Cftr *gene was disrupted or CF mutations were introduced into the murine *Cftr *gene at homologous positions. These mouse models typically exhibit a severe phenotype with intestinal obstruction that is physiologically very similar to the meconium ileus observed in humans with CF [2,3].

The transgenic *Cftr*^*TgH(neoim)Hgu *^mouse model was generated by Dorin et al. [4] using an insertional gene targeting vector to disrupt exon 10 of the *Cftr *gene in 129P2 embryonic stem cells. Unlike the *Cftr *mutants created by gene replacement, the *Cftr*^*TgH(neoim)Hgu *^mutant mice showed a surprisingly high rate of survival because low levels of wild type Cftr mRNA were produced as a result of exon skipping and aberrant splicing [5]. The original outbred *Cftr*^*TgH(neoim)Hgu *^mouse suffered from only mild intestinal obstruction, but otherwise it exhibited typical features of CF such as abnormal profiles of epithelial ion flow [4,6], mucus accumulation in gut and reproductive tracts [4] and reduced alveolar [7] and mucociliary clearance [8].

Mice from the original outbred *Cftr*^*TgH(neoim)Hgu *^mouse population with a mixed genetic background (129P2, C57BL/6, HsdOLA:MF1) were used as founder animals to establish the CF/1-*Cftr*^*TgH(neoim)Hgu *^(CF/1) and CF/3-*Cftr*^*TgH(neoim)Hgu *^(CF/3) inbred mouse strains by brother × sister mating for currently more than 40 generations [9]. When we started inbreeding, perinatal mortality was observed that peaked at the time of weaning when 8–10% of litter died of intestinal obstruction [7]. Survival was 76% at the age of 3 months for the F_8 _– F_10 _generations. CF/1 and CF/3 mice of generation 25 or more, however, did not show any increased mortality over wild type animals (pre- or post weaning) [10]. Both males and females were fertile. F_27 _– F_32 _CF/1 and CF/3 mice presented normal electrophysiology in the respiratory and intestinal epithelium, although the amounts of the expressed wild type Cftr mRNA and protein were less in the CF/3 than in the CF/1 strain [10]. Thus, inbreeding by brother × sister mating rescued from CF although the insertion mutation in exon 10 of the *Cftr *gene had been retained.

We reasoned from the close-to-normal phenotype of the CF/1 and CF/3 mice that beneficial alleles had been selected from the outbred ancestors which ameliorated the basic defect and led to close-to-normal growth, lifespan and fertility under our housing conditions. The role of secondary genetic factors for the survival of CF mice has already been demonstrated in *Cftr*^*1HSC *^knock-out mice that had been generated on a mixed genetic background by the disruption of exon 1 of the *Cftr *gene [11]. Severe intestinal obstruction led to death within the first two weeks of life or around the time of weaning in the majority of animals, but 30% of mice survived past six weeks of age. The original founder mouse was crossed with different inbred strains to generate F1 mice of different genetic backgrounds and the heterozygous F1 mice were intercrossed to produce homozygous knockout mice. The C57/BL6 cross yielded CF knock-out mice at Mendelian ratios with 25% long-term surviviors. In contrast, the numbers of CF mice of the cross with DBA/2J were much less than expected from Mendelian segregation and perinatal mortality was more than 90%. Electrophysiological studies on the mice with prolonged survival showed an upregulation of a calcium-regulated chloride conductance. This data demonstrated that the C57BL/6 background contained genetic modifier(s) associated with survival and expression of alternative chloride channels that were apparently missing in the DBA/2J background.

Since the C57BL/6 and DBA/2J backgrounds caused divergent phenotypes in CF knock-out mice, we hypothesized that the same genetic backgrounds could also be strong modifiers of the phenotype of CF mice carrying the 'leaky' *Cftr*^*TgH(neoim)Hgu *^insertion mutation. To address this issue, the insertional mutation was backcrossed from CF/3 onto the inbred backgrounds C57BL/6J and DBA/2J generating the D2.129P2(CF/3)-*Cftr*^*TgH(neoim)Hgu *^(D2-CF/3) and B6.129P2(CF/3)-*Cftr*^*TgH(neoim)Hgu *^(B6-CF/3) congenic mouse strains. These congenic mice should retain the *Cftr *linkage group of CF/3 but otherwise should be homozygous for C57BL/6J or DBA/2J alleles, respectively. Hence the comparison of the phenotype of CF/3 with that of the congenic mice could also resolve the fundamental question whether the rescue from CF in CF/3 mice was caused by secondary factor(s) in the *Cftr *linkage group and/or by loci somewhere else in the genome. Here we report on the clinical and electrophysiological features of CF/1, CF/3, the two congenic strains amd their wild type controls. The D2-CF/3 and B6-CF/3 congenic mice had retained some CF-typical signature of ion flow in the upper respiratory tract, ileum and colon, but they also showed substantial chloride secretory responses close to wild type behaviour like CF/3 in the intestine. Thus, in contrast to the observations in the *Cftr*^*1HSC *^knock-out mice, the inbred *Cftr*^*TgH(neoim)Hgu *^– derived mouse strains CF/1, CF/3, D2-CF/3 and B6-CF/3 were all able to correct the CF phenotype and presumably share the major modifier(s) of attenuation of CF symptoms.

## Results

### Genotyping

In *Cftr*^*TgH(neoim)Hgu *^mice the exon 10 of the *Cftr *gene is disrupted by the insertion of the vector pMCIneoPolyA [4]. This construct was crossed from *Cftr*^*TgH(neoim)Hgu *^transgenic CF mice into the DBA/2J and C57BL/6J backgrounds via heterozygous carriers of the mutation. The vector insert is known to be excised at low frequency in heterozygous carriers of mutant and wild type alleles [9]. Hence all CF mice of this study that were subjected to electrophysiological analysis were typed at the *Cftr *locus in order to verify their CF genotype. All typed D2-CF/3, B6-CF/3, CF/1 and CF/3 mice were carrying the pMCIneoPolyA insertion at the same integration site in exon 10 of the *Cftr *gene and were homozygous for the same intragenic *Cftr *3-marker microsatellite haplotype like their CF/3 founders [see Additional file 1]. This data demonstrated that the two congenic mouse lines shared the organization of the *Cftr *locus with that of CF/1 and CF/3 mice.

Next, the genetic background of DBA/2J, C57BL/6J and the four CF lines was compared in 26 SNPs whereby each murine chromosome was represented by one or two SNPs [see Additional file 1]. The congenic D2-CF/3 and B6-CF/3 mice shared the SNP genotypes with wild type DBA/2J and C57BL/6J mice. The exception was SNP rs3023064 located 0.5 Mbp upstream of *Cftr *where all typed B6-CF/3 mice had the same genotype as the CF/3 strain [see Additional file 1]. The shared SNP genotype profile indicated that D2-CF/3 and B6-CF/3 were congenic mouse lines. This conclusion could however have been flawed by the low informativeness of the marker set. The six inbred strains were identical in genotype for 7 of the 26 SNPs. To allow an unequivocal discrimination between the inbred strains, we selected a set of five informative microsatellites for further genotyping [see Additional file 1]. This marker set concurrently differentiated the closely related CF/1 and CF/3 and the unrelated DBA/2J, C57BL/6J and CF/3 from each other. D2-CF/3 and B6-CF/3 shared homozygous microsatellite genotypes with DBA/2J and C57BL/6J wild type, respectively. Thus, the profile of marker genotypes indicated that both D2-CF/3 and B6-CF/3 strains were congenic and were harbouring the same sequence as CF/3 mice at *Cftr *and adjacent loci.

### Growth and reproduction

Male mice had significantly higher body weight than females of the same age in all investigated mouse strains but D2-CF (Tables 1 and 2). With the exception of C57BL/6J males, body weight was similar for wild type and the corresponding congenic CF mice of the same gender indicating that the 'leaky' insertion mutation [5] did not significantly affect growth. Inbred CF/1 and CF/3 mice were heavier than the congenic CF mice of the same age and gender in accordance with the lower body weight of wild type inbred lines DBA/2J and C57BL/6J compared to outbred HsdOla:MF1 (Tables 1 and 2). Body weight of CF/1 and CF/3 mice of both sexes was similar to that of DBA/2J males, but higher than that of DBA/2J females. Similarly, CF/1 females were heavier than C57BL/6 females. In summary, gender and genetic background were more important than the *Cftr*^*TgH(neoim)Hgu *^mutation in shaping body weight.

**Table 1:** Body weights of the investigated mice.

**Strain**	**Age (days, mean ± SD)**	**Generation**	**Number of mice**	**Weight (g)**
**DBA/2J**	118.4 ± 8.3	F 167-F 169	9 ♀	21.7 ± 0.5
			9 ♂	27.6 ± 2.6
**C57BL/6J**	113.7 ± 8.4	F 166-F 170	9 ♀	21.2 ± 2.0
			9 ♂	31.7 ± 2.6
**HsdOla:MF1**	140.4 ± 2.1*	no data	9 ♀	37.7 ± 4.8
			9 ♂	43.4 ± 5.1
**D2.129P2(CF/3)-*Cftr*^*TgH(neoim)Hgu*^**	114.6 ± 5.7	N10F 9-F 12	9 ♀	20.8 ± 2.7
			9 ♂	23.7 ± 3.0
**B6.129P2(CF/3)-*Cftr*^*TgH(neoim)Hgu*^**	107.8 ± 8.9	N12F 8-F 12	9 ♀	20.4 ± 0.9
			9 ♂	26.1 ± 2.1
**CF/1-*Cftr*^*TgH(neoim)Hgu*^**	111.9 ± 8.7	F 27-F 40	9 ♀	25.4 ± 1.8
			9 ♂	29.9 ± 3.3
**CF/3-*Cftr*^*TgH(neoim)Hgu*^**	122.1 ± 8.1	F 27-F 40	9 ♀	24.4 ± 2.9
			9 ♂	31.3 ± 2.5
**DBA/2J^§^**	112		♀	Jaxpheno1: 24.4
				Jaxpheno2: 27.0
				Jaxpheno5: 24.9
			♂	Jaxpheno1: 28.6
				Jaxpheno2: 29.2
				Jaxpheno5: 28.9
**C57BL/6J^§^**	112		♀	Jaxpheno1 21.9
				Jaxpheno2: 22.0
				Jaxpheno5: 22.1
			♂	Jaxpheno1: 29.1
				Jaxpheno2: 30.5
				Jaxpheno5: 30.0

* The body weight of HsdOla:MF1 mice by the age of 120 ± 9 days had been determined in a previous study [7] to be 31 ± 2 g.

^§^Data were obtained from the Mouse Phenome Database of the Jackson Laboratory, Bar Harbor, Maine, USA [33].

**Table 2:** Significance of differences in mean body weight differentiated by strain and gender

		**DBA/2J**		**C57BL/6J**		**HsdOla:MF1**		**D2-CF/3**		**B6-CF/3**		**CF/1**		**CF/3**	
		♂	♀	♂	♀	♂	♀	♂	♀	♂	♀	♂	♀	♂	♀
**DBA/2J**	♂		***	*	**	na	na	ns	*	ns	***	ns	ns	ns	ns
	♀			***	ns	na	na	ns	ns	**	*	***	**	***	*
**C57BL/6J**	♂				***	na	na	**	***	**	***	ns	**	ns	**
	♀					na	na	ns	ns	*	ns	**	*	***	ns
**Hsd Ola: MF1**	♂						*	na	na	na	na	na	na	na	na
	♀							na	na	na	na	na	na	na	na
**D2-CF/3**	♂								ns	ns	ns	*	ns	**	ns
	♀									*	ns	**	*	***	ns
**B6-CF/3**	♂										***	ns	ns	*	ns
	♀											***	**	***	*
**CF/1**	♂												*	ns	*
	♀													**	ns
**CF/3**	♂														**
	♀														

*, *P *< 0.05; **, *P *< 0.01; ***, *P *< 0.001; ns, not significant na, not applicable because of the higher age of HsdOla:MF1 mice

The inbred CF/1, CF/3 and B6-CF/3 mice were fertile in the homozygous CF state. D2-CF/3 females, however, were not fertile. Hence, the line was propagated by crossing D2-CF/3 males with female heterozygous carriers of the *Cftr*^*TgH(neoim)Hgu *^mutation. Tables 3 and 4 provide information about the reproductive fitness of the investigated strains. Wild type C57BL/6 mice generated the highest number of offspring. Litter size of the congenic B6-CF/3 strain was comparable, but the number of litters per breeding pair was lower than that of the wild type strain (*P *< 0.01). CF/1 and CF/3 mice produced the lowest number of litters. Litter size, however, was normal. CF/3 mice generated more progeny per litter than DBA/2J and as many as C57BL/6J mice (Table 3).

**Table 3:** Fertility of mouse strains

**Strain**	**Generation**	**No of breeding pairs**	**No of litters per breeding pair**	**Litter size**
			
			Median (inner quartiles; range)	
DBA/2J	F 167-F 169	7	4 (3–7; 1–14)	4 (2–5; 1–8)
C57BL/6J	F 166-F 170	68	6 (5–8; 2–10)	6 (4–8; 1–11)
D2.129P2(CF/3)-*Cftr*^*TgH(neoim)Hgu*^	N10F 9-F 12	19	4 (3–7; 1–10)	4 (2–5; 1–10)
B6.129P2(CF/3)-*Cftr*^*TgH(neoim)Hgu*^	N12F 8-F 12	21	4 (3–5; 1–7)	5 (3–7; 1–11)
CF/1-*Cftr*^*TgH(neoim)Hgu*^	F 27-F 40	106	3 (2–4; 1–10)	5 (3–6; 1–10)
CF/3-*Cftr*^*TgH(neoim)Hgu*^	F 27-F 40	93	3 (2–3; 1–8)	6 (4–7; 1–10)

**Table 4:** Significance of interstrain differences of fertility (litter size and number of litters per breeding pair)

**No. of litters per breeding pair**						
	DBA/2J	C57BL/6J	D2-CF/3	B6-CF/3	CF/1	CF/3

DBA/2J		ns	ns	ns	***	***
C57BL/6J	ns		ns	**	***	***
D2-CF/3	ns	**		ns	***	***
B6-CF/3	ns	ns	ns		ns	*
CF/1	ns	***	*	ns		ns
CF/3	*	ns	***	ns	**	

**Litter size**						

*, *P *< 0.05; **, *P *< 0.01; ***, *P *< 0.001; ns, not significant

Survival rates beyond weaning were 81% and 91% for wild-type DBA/2J and C57BL/6J, both 87% for the two congenic strains D2-CF/3 and B6-CF/3, 86% for CF/1 and 91% for CF/3. Under our housing conditions death rates during the perinatal and weaning periods were not higher than in wild type mice for all four inbred CF mouse lines.

### Electrophysiology

**Figure 1:**
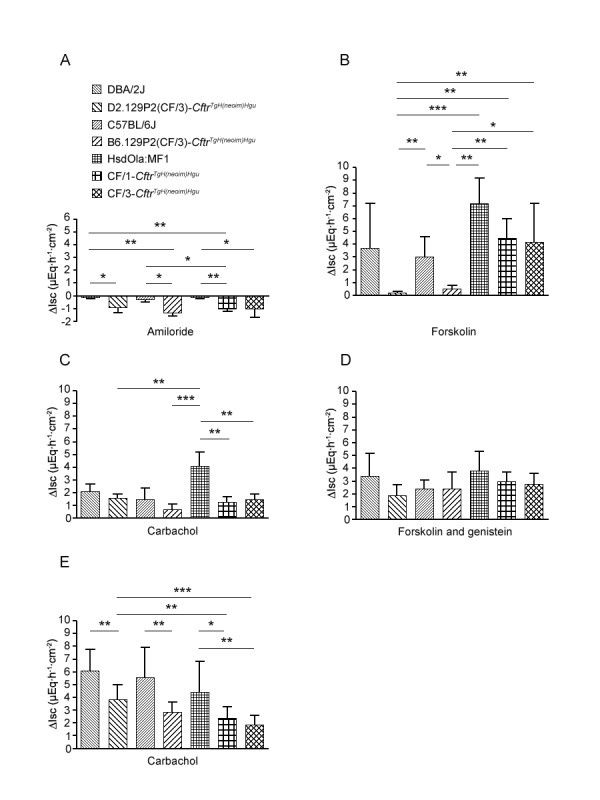
Short circuit current measurements in the epithelia of the upper respiratory tract (A, B, *n *= 4 per mouse line), ileum (C, D, *n *= 4) and colon epithelium (E, *n *= 10) in inbred *Cftr*^*TgH(neoim)Hgu *^mouse strains and their cognate wild type controls. ΔIsc represents the change in short circuit current from baseline after addition of either amiloride (A), forskolin (B), forskolin + genistein (D) and carbachol (C, E). * *P *< 0.05; ** *P *< 0.01; *** *P *< 0.001.

Aberrant epithelial ion flow is the hallmark of the basic defect in CF [1]. We investigated the secretory responses of nasal, ileal and colon epithelia upon exposure to secretagogues and channel blockers in the four inbred *Cftr*^*TgH(neoim)Hgu *^strains and their corresponding wild type controls. The outcome of the Ussing chamber measurements is summarized in the histograms of Figure 1.

CF nasal epithelium was exposed to the sodium channel blocker amiloride (Fig. 1A). Whereas all wild type mouse strains showed a minute response to amiloride, nasal tissues of all four CF mouse strains responded with a decrease of the short circuit current within the range of 1 μEqh^-1^cm^-2^. Next, forskolin was added which activates adenylate cyclase, production of cAMP and subsequent opening of cAMP-dependent chloride conductances. Forskolin evoked a stronger chloride secretory response in wild type than in the corresponding CF nasal epithelium in accordance with the expectation that forskolin should elicit a larger luminal flow of chloride in Cftr-proficient than in Cftr-compromised mice (Fig. 1B). This difference was particularly pronounced in the two congenic CF strains, although – due to the large scatter of values of individual DBA/2J mice – it was only significant for the comparison between C57BL/6J and B6-CF/3 mice. The genetic background was as least as important as the non-CF vs. CF state for luminal chloride flow. The CF strains CF/1 and CF/3, for example, showed a similar chloride secretory response as the non-CF DBA/2J and C57BL/6J mice.

Next, the chloride secretory responses upon stimulation with carbachol or with the cAMP agonist forskolin and the phosphodiesterase inhibitor genistein were measured in the mouse ileum which is the site of the most profound pathology in most CF mouse models, namely the fatal intestinal obstruction syndrome [2,3,11]. This complication was absent in all four strains CF/1, CF/3, B6-CF/3 and D2-CF/3. The proximal intestine is known to express numerous anion transporters, exchangers and channels, of which CFTR is the major respondent upon activation with forskolin [12]. The addition of forskolin and genistein stimulated statistically indistinguishable short circuit currents in the ileum of all seven tested mouse strains (Fig. 1D). The interindividual response within a strain was more variable than the mean interstrain difference suggesting that at least in the ileum all four CF strains produced sufficient Cftr and/or other chloride conductances to mount a normal or close-to-normal chloride secretory response.

Carbachol was the other agonist to stimulate chloride secretion. Carbachol initiates the cholinergic activation of chloride secretion by increasing the intracellular Ca^2+ ^concentration through stimulation of Ca^2+ ^influx and mobilization of intracellular Ca^2+ ^stores [13]. This causes Ca^2+ ^dependent K^+ ^efflux, which acts as the electrogenic driving force for apical chloride secretion [14]. Moreover, carbachol activates CFTR in the apical membrane by increasing the formation of diacylglycerol, thus stimulating the protein kinase C – dependent signaling pathway [15]. A large transient chloride secretory response upon addition of carbachol, i.e. an increase of Isc, is characteristic for intestinal epithelia and is caused mainly by CFTR-mediated chloride secretion [16].

The mean chloride secretory response of the tested mice to serosal application of carbachol was 1.3- to 3-fold stronger in wild-type than in their corresponding *Cftr*^*TgH(neoim)Hgu *^strain(Fig. 1C). However, pairwise comparisons revealed significantly larger Isc only between the outbred HsdOla:MF1 and the four CF strains (Fig. 1C). The ileal epithelium of CF/1 and CF/3 showed a similar Isc response as that of wild type C57BL/6J mice. In other words, genetic loci other than *Cftr *influenced the response to carbachol.

The high levels of Cftr mediated chloride secretion in the ileum of the congenic CF mice were unexpected, although it corresponded to their uniform mild phenotype. To address the issue if a substantial carbachol-induced chloride secretory response was a general feature of the intestine of congenic mice, we also measured the carbachol response in the colon. The number of animals in each group was increased in order to unequivocally answer the question whether or not isogenic non-CF and *Cftr*^*TgH(neoim)Hgu *^mouse strains could be differentiated by their transient chloride secretory response to carbachol which is the basis for the diagnosis of CF in intestinal current measurements in humans [17,18]. Moreover the protocol was modified so that the carbachol response should almost exclusively be caused by Cftr-mediated chloride secretion. The proximal and distal specimens from the colon ascendens were preincubated with amiloride, TEA and BaCl_2 _in order to block the apical Na^+ ^and K^+ ^conductances. Subsequently, the tissues were preincubated with DIDS prior to carbachol addition. DIDS does not affect the Cftr channel from the extracellular side at physiological pH, but it blocks other chloride conductances such as the CaCC and ORCC [19,20].

Taking a size of ten animals per group and following the modified protocol, the change of short circuit current in colonic epithelium induced by carbachol was significantly lower in all CF strains than in their wild type controls (Fig. 1E), although the values recorded for individual CF and non-CF mice were overlapping. Mean Isc values of the D2-CF/3 and the B6-CF/3 strain were 63% and 51% of those of DBA/2J and C57BL/6J mice, respectively. In contrast to the findings in the nasal and ileal epithelia, the mice with the DBA/2J and C57BL/6J backgrounds mounted stronger chloride secretory responses in the colon to carbachol than HsdOla:MF1 and the derived CF/1 and CF/3 mice. Carbachol-stimulated chloride secretion was within the same range in colon and ileum of the three latter strains with MF1 background. In contrast, the response was 4-fold higher in the colon than in the ileum of the other four investigated strains (Figs. 3C, 3E). Hence, the genetic backgrounds divergently influenced absolute and relative levels of Cftr-mediated chloride secretion in the proximal and distal intestine.

## Discussion

The analysis of four inbred *Cftr*^*TgH(neoim)Hgu *^mouse strains and their wild type controls allowed us to address two basic complementary aspects of genotype-phenotype association studies.

First, a CF congenic strain should only differ at the *Cftr *chromosomal region from the wild type, and hence the comparison between CF and non-CF mice should dissect the role of the *Cftr*^*TgH(neoim)Hgu *^mutation on murine phenotype in this particular genetic background. The CF basic defect of hyperresponsiveness to amiloride and lower chloride secretory responses to carbachol and cAMP agonists [1] was common for all *Cftr*^*TgH(neoim)Hgu *^mutant mice. Severe macroscopic pathology or CF-typical clinical features, however, were not observed. This very mild phenotype that particularly was not anticipated for the congenic strains with the DBA/2J or C57BL/6J backgrounds, is in sharp contrast to the severe phenotype of CF mice that carry null mutations in the same genetic backgrounds [21,22].

Second, the comparison of numerous *Cftr*^*TgH(neoim)Hgu *^strains should reveal the role of non-*Cftr *genes on the phenotype of CF mice with the same disease-causing mutation. The most remarkable findings were strain-specific signatures of weight gain and of secretagogue-induced ion flow and the reduced fertility of female D2-CF/3 mice not seen in any of the other three inbred *Cftr*^*TgH(neoim)Hgu *^strains. Insufficient weight gain, infertility and aberrant epithelial ion flow are key features of CF [1]. By using our inbred *Cftr*^*TgH(neoim)Hgu *^mouse models, the murine loci could be mapped that account for these strain-specific differences in phenotypic traits that are relevant for human CF disease. The comparison of the same *Cftr *mutation in different genetic backgrounds has already been employed by Haston and colleagues to identify modifier loci of the CF phenotype in lung [22], intestine [23] and body weight [24] by quantitative trait loci mapping of C57BL/6J and BALB/c mice carrying the *Cftr*^*tm1UNC *^null mutation.

The four inbred *Cftr*^*TgH(neoim)Hgu *^mouse strains exhibited a very mild CF phenotype under the standardized living conditions in our animal facility as indicated by close-to-normal growth and lifespan and a substantial chloride secretory response to carbachol and forskolin of 15 – 100% of wild-type levels. Weight gain and survival of the CF/1 and CF/3 mice continuously improved with the number of brother × sister matings that had been started from four outbred littermates [10]. Whereas the initial outbred transgenic *Cftr*^*TgH(neoim)Hgu *^mutant mice exhibited typical pathophysiological features of CF in gut, lung and reproductive tract [4,6,7], the CF/1 and CF/3 mice of generation 20 or more ameliorated the basic defect and showed normal survival [10]. This favourable phenotype was retained when the *Cftr*^*TgH(neoim)Hgu *^insertional mutation was introduced from inbred CF/3 mice into the C57Bl/6J and DBA/2J backgrounds. Hence, we would like to conclude that the close-to-normal phenotype of the four *Cftr*^*TgH(neoim)Hgu *^strains results from positive modifier(s) located in sequences shared by all mice, namely *Cftr *and hitchhiking loci in cis, although we cannot exclude that some CF/3 sequences elsewhere in the genome were under such a strong positive selection during the production of the congenic mice that they had been retained in the D2-CF/3 and B6-CF/3 strains.

Although the typical clinical aspects of CF such as underweight and poor survival were not seen in the four inbred *Cftr*^*TgH(neoim)Hgu *^mouse lines, we nevertheless observed some CF-typical features in our clinically inconspicuous mice. The functional assessment of the upper respiratory epithelium uncovered a CF-typical electrophysiological phenotype. Besides a lower response to forskolin, a strong hyperresponsiveness to amiloride was noted in all *Cftr*^*TgH(neoim)Hgu *^strains. Previous semiquantitative immunoblot analysis of CF mouse tissue indicated that about 10–15 % of Cftr wild type protein levels are sufficient to normalize Cftr – mediated chloride secretion [10,25], but do not correct the hyperabsorption of sodium ions. This finding agrees with the outcome of *CFTR *gene transfer into human CF airway epithelial cells that subnormal levels of CFTR restore cAMP-mediated chloride secretion, but that the normalization of sodium hyperabsorption follows a linear dose-response relationship [26,27,32]. In summary, the basic electrophysiological defect of CF was apparent in all tissue comparisons between CF and non-CF strains with the same genetic background, although it was more visible in the nose than in ileum and colon.

The most unexpected finding of our study was the clinically asymptomatic phenotype of the congenic D2-CF/3 and B6-CF/3 mice. It seemed as if the pathophysiological consequences of the *Cftr *insertion mutation had been neutralized by beneficial modifiers. However, we cannot yet exclude that the mild phenotype is at least partially based upon the favourable living conditions in the animal facility with its unrestricted access to food and prevention from pathogens. Fitness differences between congenic CF and non-CF mice may become apparent in the wild. Future studies of how *Cftr*^*TgH(neoim)Hgu *^mouse strains can cope with CF-typical stress conditions could resolve this issue.

## Conclusion

Brother × sister matings over 40 generations switched the mild phenotype of the original outbred *Cftr*^*TgH(neoim)Hgu *^mouse to close-to-normal clinical phenotypes of inbred CF/1 and CF/3 mice. Transfer of the mutated *Cftr *locus into the standard backgrounds DBA/2J and C57BL/6J retained this very mild phenotype. CF-typical bioelectrics were still seen in the congenic CF mice and allowed a differentiation between CF and non-CF states, although not at the level of the individual animal. With the exception of fertility of D2-CF/3 females, residual chloride secretion was sufficiently large for having no clinical consequences.

The mild phenotype of CF/1, CF/3, B6-CF/3 and D2-CF/3 mice indicates that loci shared between the inbred strains, most likely the *Cftr *linkage group, contain the major modifier(s) of attenuation of CF symptoms.

## Methods

### Experimental animals

Offspring (generation F4) of the original *Cftr*^*TgH(neoim)Hgu *^mouse population with divergent genetic backgrounds originating from the 129/Sv, C57BL/6 and HsdOla:MF1 strains [4] were used to generate the CF/1 and CF/3 inbred mouse strains by brother × sister mating at the Central Animal Facility of the Hannover Medical School [9]. Congenic mouse strains were generated on DBA/2J and C57BL/6J backgrounds by using *Cftr*^*TgH(neoim)Hgu *^mice as a donor strain establishing the D2-CF/3 and B6-CF/3 strains [9,28] by speed congenic production [29].

All experiments were approved by the local government as well as by the local Institutional Animal Care and Research Advisory Committee. All mice were bred in the Central Animal Facility of the Hannover Medical School, except for the HsdOla:MF1 strain. Mice of this strain had been bred by Harlan Winkelmann GmbH (Borken, Germany) and were shipped to the Central Animal Facility of the Hannover Medical School four weeks before the beginning of the experiments.

The C57BL/6J strain was kept under specified pathogen-free conditions. All other mouse strains were kept in individually ventilated cages. The temperature was 21°C, the relative humidity 55 ± 5%. The C57BL/6J strain was fed an irradiated standard chow (ssniff R-Z V1324-300) and autoclaved water ad libitum. The other mouse strains were fed a different irradiated standard chow (altromin 1314) and filtered water also ad libitum. Litter size and number of litters were recorded for all breeding pairs housed at Hannover Medical School.

### Genotyping of mice

High molecular weight DNA was isolated from murine liver tissue based on the protocol by Gross-Bellard et al. [30]. Mice were genotyped by a low density SNP scan (Kbiosciences, Hoddesdon, England), microsatellites and PCR spanning the integration sites of the pMC1neoPolyA vector in exon 10 of *Cftr *[9]. Microsatellites were amplified by PCR from genomic DNA, separated by direct blotting electrophoresis [9] and evaluated as outlined by Mekus et al. [31].

During the generation of the congenic strains, the insertion of the vector was verified for all litters by Southern blot hybridizations [4,9]. This control was necessary because the spontaneous excision of the vector has been observed at a frequency of 4–5% in heterozygous carriers of the insertion mutation [9].

### Short circuit current measurements

Freshly excised mouse ileum, colon and nasal epithelium of CF/1, CF/3, D2-CF/3 and B6-CF/3 homozygous animals were used for short circuit current (Isc) measurements and compared with wild type controls. Experiments were performed at 37°C. The perfusion solution was saturated with 95% O_2 _and 5% CO_2_, pH 7.4.

Modified Meyler's solution (128 mM NaCl, 4.7 mM KCl, 1.3 mM CaCl_2_, 1.0 mM MgCl_2_, 20.2 mM NaHCO_3_, 0.4 mM NaH_2_PO_4_, 0.33 mM Na_2_HPO_4_, 10 mM HEPES) was used for the measurements on ileum and nasal epithelium, and Krebs-Henseleit solution (113.6 mM NaCl, 5.4 mM KCl, 1.2 mM CaCl_2_, 1.2 mM MgCl_2_, 0.6 mM NaH_2_PO_4_, 2.4 mM Na_2_HPO_4_, 21.0 mM NaHCO_3_, 10.0 mM glucose and 19.7 mM mannitol) was used for the measurements on colon epithelium.

Mouse ileum or colon was excised under hypnorm/diazepam anaesthesia and reverted on a plastic rod. The muscle layer was cut longitudinally using a blunt razor blade and was stripped of fat manually. The stripped tissue was mounted in a holder with the mucosal side up (exposed tissue area 0.2 or 0.5 cm^2^). After insertion of the holder into the Ussing chamber the tissue was allowed to recover for 10–20 min and to reach a stable baseline. The tissues were then short-circuited for the whole experimental period.

In case of ileum tissue specimens, glucose (10 mM) and indomethacin (10 μM) were added to the serosal side. After equilibrium, the following compounds were added consecutively to the mucosal (M) or serosal (S) side of the tissue: Forskolin (10 μM, S), genistein (100 μM, M+S) and carbachol (200 μM, S). All compounds were present throughout the experiment. Experiments on the colon were performed with a proximal and a distal specimen of colon ascendens in each wild type and CF mouse. After equilibration the following drugs were sequentially added: amiloride (100 μM, M), TEA (tetraethylammonium, 5 mM, M), Ba^2+ ^(BaCl_2_, 1 mM, M), DIDS (4,4'-diisothiocyanostilbene-2,2'-disulfonic acid, 200 μM, M) and carbachol (100 μM, S).

Mouse nasal epithelium was isolated as described by Grubb et al. [32]. In brief, the mice were sacrificed by cervical dislocation and the skin of the head was peeled back in order to reach the underlying paired nasal bones. These were removed and the two sheets of the nasal epithelia, separated by the septum, were isolated independently. The sheets of epithelia were mounted immediately between the Ussing chambers (exposed area 1.13 mm^2^). The chambers were filled with gassed modified Meyler's solution supplemented with glucose (10 mM) and indomethacin (10 μM) and the short circuit measurements were started. After equilibration amiloride (10 μM, M) was added followed by forskolin (10 μM, S, after stabilization of the current).

### Statistics

Data are presented as means ± standard deviation (SD), *n *represents the number of mice. Unpaired t-test was used for comparisons between two groups. One-way ANOVA followed by a post hoc Tukey's test was applied for comparisons within one group. *P*-values < 0.05 were considered to be significant. The statistical analysis was performed using GraphPad Prism version 4.00 for Windows.

## Abbreviations

CACC, calcium-activated chloride channel

CF, cystic fibrosis

CFTR, cystic fibrosis transmembrane conductance regulator

DIDS, 4,4'-diisothiocyanostilbene-2,2'-disulfonic acid

HEPES, 4-(2-hydroxyethyl)-1-piperazineethanesulfonic acid

Isc, short circuit current

ORCC, outwardly rectifying chloride channel

PBS, phosphate buffered saline

SD, standard deviation

SNP, single nucleotide polymorphism

TEA, tetraethylammonium

## Authors' contributions

BTo, FS, SJ, DW and NC performed genotyping. BTo, MW, AB and MB carried out short circuit current measurements. FS set up all PCR-related methodology and quality control and assisted with the interpretation of all molecular genetic data. MD and DW participated in the supervision of animal breeding. SLM assisted with the planning and interpretation of Ussing chamber measurements. HJH designed and supervised all animal breeding. HRDJ, HJH, GB and BTü conceived the study and participated in the design of experiments and results analysis. BTo and BTü drafted the manuscript. All authors read and approved the final manuscript.

## Supplementary Material

Additional file 1 CF mouse genotyping. A. Map localisation of SNPs. B. Marker genotypes of 26 SNPs and 8 microsatellites in the DBA/2J, D2.129P2(CF/3)-*Cftr*^*TgH(neoim)Hgu*^, C57BL/6J and B6.129P2(CF/3)-*Cftr*^*TgH(neoim)Hgu*^, CF/1-*Cftr*^*TgH(neoim)Hgu *^and CF/3-*Cftr*^*TgH(neoim)Hgu *^mouse strains. Click here for file
