# Supplementary material for: Very mild disease phenotype of congenic CftrTgH(neoim)Hgu cystic fibrosis mice
Source: BMC Genet. 2008 Apr 9;9:28. doi: 10.1186/1471-2156-9-28 (PMC2323021; doi:10.1186/1471-2156-9-28)
Supplement: Additional file 1 — CF mouse genotyping. A. Map localisation of SNPs. B. Marker genotypes of 26 SNPs and 8 microsatellites in the DBA/2J, D2.129P2(CF/3)-CftrTgH(neoim)Hgu, C57BL/6J and B6.129P2(CF/3)-CftrTgH(neoim)Hgu, CF/1-CftrTgH(neoim)Hgu and CF/3-CftrTgH(neoim)Hgu mouse strains. [file 1471-2156-9-28-S1.doc]

Additional File 1. CF mouse genotyping

A. Map localisation of SNPs

|  | SNP | **Chromo-some No.** | **Map position*** | **rs number** |
| --- | --- | --- | --- | --- |
|  | **M-22381_169_2** | 1 | 89100712 | rs3022825 |
|  | **M-09011_1** | 2 | 37606770 | rs3022883 |
|  | **M-02707_1** | 3 | 37299427 | rs3022953 |
|  | **M-01609_1** | 4 | 19965760 | na |
|  | **M-05233_3** | 5 | 24391318 | rs3023039 |
|  | **M-02187_2** | 5 | 7163492 | rs3023034 |
|  | **M-02094_1** | 6 | 17673652 | rs3023064 |
|  | **M-05782_1** | 7 | 3793412 | rs3023106 |
|  | **M-11559_2** | 8 | 14759100 | rs3023177 |
|  | **M-04819_1** | 9 | 100882655 | rs3023226 |
|  | **M-05537_1** | 9 | 28930591 | rs3023203 |
|  | **M-09526_2** | 10 | 8657805 | rs3089984 |
|  | **M-05799_1** | 10 | 87228038 | rs3089109 |
|  | **M-05727_1** | 11 | 20853304 | rs3023251 |
|  | **M-08924_1** | 11 | 8402197 | rs3088673 |
|  | **M-07403_3** | 12 | 30739697 | rs3023342 |
|  | **M68896_151_1** | 13 | 21380825 | rs3023381 |
|  | **M-05495_2** | 13 | 44496918 | rs3023382 |
|  | **M-07251_1** | 14 | 21661996 | rs3091048 |
|  | **M-07646_1** | 15 | 32625942 | rs3091174 |
|  | **M-01322_2** | 16 | 88158279 | rs3023436 |
|  | **M-04659_1** | 17 | 34330736 | na |
|  | **AF027865_1** | 17 | 34330565 | rs3023450 |
|  | **M-09844_1** | 18 | 68285062 | rs3089349 |
|  | **M-02162_1** | 19 | 20492437 | rs3023481 |
|  | **M-05810_1** | X | 66915814 | rs3089604 |

*Map position according to the Genbank entry. Please note that map positions were not consistent between the Genbank and Celera entries (February 11, 2008).

na, not available

B. Marker genotypes of 26 SNPs and 8 microsatellites in the DBA/2J, D2.129P2(CF/3)-*Cftr^TgH(neoim)Hgu^*, C57BL/6J and B6.129P2(CF/3)-*Cftr^TgH(neoim)Hgu^*, CF/1-*Cftr^TgH(neoim)Hgu^* and CF/3-*Cftr^TgH(neoim)Hgu^* mouse strains.

| **Markers** | DBA/2J | **D2.129P2(CF/3)-*Cftr^TgH(neoim)Hgu^ **** | **C57BL/6J** | **B6.129P2(CF/3)-*Cftr^TgH(neoim)Hgu^*** | **CF/1-*Cftr^TgH(neoim)Hgu^*** | **CF/3-*Cftr^TgH(neoim)Hgu^*** |
| --- | --- | --- | --- | --- | --- | --- |
| SNPs | | | | | | |
| **M-22381_169_2** | C:C | C:C | T:T | T:T | C:C | C:C |
| **M-09011_1** | C:C | C:C | A:A | A:A | C:C | C:C |
| **M-02707_1** | C:C | C:C | T:T | T:T | T:T | T.T |
| **M-01609_1** | T:T | T:T | C:C | C:C | T:T | T:T |
| **M-05233_3** | G:G | G:G | A:A | A:A | A:A | A:A |
| **M-02187_2** | T:T | T:T | G:G | G:G | G:G | G:G |
| **M-02094_1** | T:T | T:T | G:G | T:T | T:T | T:T |
| **M-05782_1** | G:G | G:G | T:T | T:T | T:T | T:T |
| **M-115592_2** | A:A | A:A | C:C | C:C | A:A | A:A |
| **M-04819_1** | C:C | C:C | T:T | T:T | T:T | T:T |
| **M-05537_1** | A:A | A:A | A:A | A:A | A:A | A:A |
| **M-09526_2** | T:T | T:T | T:T | T:T | G:G | G:G |
| **M-05799_1** | G:G | G:G | G:G | G:G | G:G | G:G |
| **M-05727_1** | C:C | C:C | T:T | T:T | C:C | T:T |
| **M-08924_1** | A:A | A:A | A:A | A:A | A:A | A:A |
| **M-07403_3** | C:C | C:C | T:T | T:T | C:C | C:C |
| **M68896_151_1** | T:T | T:T | C:C | C:C | C:C | C:C |
| **M-05495_2** | T:T | T:T | T:T | T:T | T:T | T:T |
| **M-07251_1** | T:T | T:T | T:T | T:T | T:T | T:T |
| **M-07646_1** | A:A | A:A | G:G | G:G | A:A/G:A/G:G | A:A |
| **M-01322_2** | T:T | T:T | A:A | A:A | A:A | A:A |
| **M-04659_1** | A:A | A:A | A:A | A:A | A:A | A:A |
| **AF027865_1** | C:C | C:C | T:T | T:T | T:T | T:T |
| **M-09844_1** | G:G | G:G | ? | G:G | G:G | G:G |
| **M-02162_1** | G:G | G:G | G:G | G:G | A:A | G:G |
| **M-05810_1** | C:C | C:C | T:T | T:T | C:C | C:C |
|  | | | | | | |
| **Microsatellites outside the *Cftr* locus** | | | | | | |
| **D3Mit320** | 19/19 | 19/19 | 24/24 | 24/24 | 20/20 | 20/20 |
| **D3Mit292** | 19/19 | 19/19 | 20/20 | 20/20 | 19/19 | 20/20 |
| **MitBTo1** | 20/20 | 20/20 | 20/20 | 20/20 | 22/22 | 20/20 |
| **MitBTo2** | 25/25 | 25/25 | 20/20 | 20/20 | 20/20 | 20/20 |
| **MitBTo3** | 16/16 | 16/16 | 22/22 | 22/22 | 22/22 | 20/20 |
|  | | | | | | |
| **Intragenic microsatellites at the *Cftr* locus** | | | | | | |
| **D6NC3** | 20/20 | 20/20 | 20/20 | 20/20 | 20/20 | 20/20 |
| **D6Mit236** | 20/20 | 20/20 | 20/20 | 20/20 | 20/20 | 20/20 |
| **D6NC5** | 20/20 | 20/20 | 20/20 | 20/20 | 20/20 | 20/20 |

Genotypes that differ between wild type and congenic CF strains are highlighted in grey.

10 CF/1-*Cftr^TgH(neoim)Hgu^*, 10 CF/3-*Cftr^TgH(neoim)Hgu^*, 14 D2.129P2(CF/3)-*Cftr^TgH(neoim)Hgu^* and 10 B6.129P2(CF/3)-*Cftr^TgH(neoim)Hgu^* mice taken from several litters.

The number 20 was arbitrarily assigned to the microsatellite alleles of strain CF/3. The difference of repeats is counted, for example, a 25-allele carries five di-, tri- or tetranucleotide repeats more than the reference allele and a 16-allele carries 4 repeats less than the reference allele.
